# Associations between inflammation and striatal dopamine D2-receptor availability in aging

**DOI:** 10.1186/s12974-025-03355-0

**Published:** 2025-01-30

**Authors:** Vanessa Crine, Goran Papenberg, Jarkko Johansson, Carl-Johan Boraxbekk, Anders Wåhlin, Ulman Lindenberger, Martin Lövdén, Katrine Riklund, Lars Bäckman, Lars Nyberg, Nina Karalija

**Affiliations:** 1 Department of Medical and Translational Biology, Umeå university, Umeå, 901 87 Sweden; 2 Umeå Center for Functional Brain Imaging (UFBI), Umeå University, Umeå, Sweden; 3 Aging Research Center, Karolinska Institute and Stockholm University, Stockholm, Sweden; 4 Department of Diagnostics and Intervention, Umeå University, Umeå, Sweden; 5 Institute for Clinical Medicine, Faculty of Medical and Health Sciences, University of Copenhagen, Copenhagen, Denmark; 6 Institute of Sports Medicine Copenhagen (ISMC), Department of Neurology, Copenhagen University Hospital Bispebjerg, Copenhagen, Denmark; 7 Center for Lifeorgdivision Psychology, Max Planck Institute for Human Development, Berlin, Germany; 8 Max Planck UCL Centre for Computational Psychiatry and Ageing Research, Berlin, Germany and London, UK; 9 Department of Psychology, University of Gothenburg, Gothenburg, Sweden; 10 Department of Applied Physics and Electronics, Umeå University, Umeå, Sweden

**Keywords:** Inflammation, Dopamine D2-receptor availability, Positron emission tomography, White-matter lesions, Aging

## Abstract

**Background:**

Normal brain aging is associated with dopamine decline, which has been linked to age-related cognitive decline. Factors underlying individual differences in dopamine integrity at older ages remain, however, unclear. Here we aimed at investigating: (i) whether inflammation is associated with levels and 5-year changes of in vivo dopamine D2-receptor (DRD2) availability, (ii) if DRD2-inflammation associations differ between men and women, and (iii) whether inflammation and cerebral small-vessel disease (white-matter lesions) serve as two independent predictors of DRD2 availability.

**Methods:**

Analyses were performed in a sample of healthy adults > 60 years assessed at two measurement occasions separated by 5 years. At both occasions, DRD2 availability was estimated by ^11^C-raclopride PET, and white-matter lesions by MRI. Inflammation was assessed by two C-reactive protein-associated DNA methylation scores at study baseline.

**Results:**

Individuals with higher DNA methylation scores at baseline showed reduced striatal DRD2 availability. An interaction was found between DNA methylation scores and sex in relation to striatal DRD2 availability, such that associations were found in men but not in women. DNA methylation scores at study entrance were not significantly associated with 5-year striatal DRD2 decline rates. No significant association was found between DNA methylation scores and white-matter lesions, but higher scores as well as higher lesion burden were independently associated with reduced striatal DRD2 availability in men.

**Conclusions:**

These findings suggest negative associations between one proxy of inflammation and DRD2 availability in older adults, selectively for men who had higher DNA methylation scores. Future studies should investigate other inflammatory markers in relation to dopamine integrity.

**Supplementary Information:**

The online version contains supplementary material available at 10.1186/s12974-025-03355-0.

## Background

The age-sensitivity of the dopamine system is suggested to give rise to negative consequences in older populations, including cognitive disturbances and depression [1, 2]. Numerous cross-sectional imaging studies show that striatal dopamine markers, e.g., receptors, are reduced with increasing age [3]. This finding is now supported by longitudinal data, showing within-person striatal dopamine D2-receptor (DRD2) reductions in healthy, older adults across five years of aging [4]. While individual differences in dopamine markers are well documented, both in terms of baseline levels and rates of change [4–6], little is known about factors accounting for such differences.

Manifestations of cerebral small-vessel disease (white-matter lesions) have been associated with individual differences in dopamine integrity in cross-sectional designs [7–9]. Longitudinal work further shows that faster progression of lesion volumes is associated with more rapid DRD2 loss across 5 years of aging [4]. Inflammation is suggested to constitute yet another potential driver of dopaminergic decline [2]. While transient inflammation is protective during an infection or trauma, aging-related chronic inflammation is characterized by an unregulated immune response manifested by increased levels of inflammatory markers [10–12]. This unresolved systemic low-grade inflammation, referred to as inflammaging [13], may in turn give rise to age-related malfunctions [14, 15], including cardiovascular disease [16–19] and exacerbated cognitive decline [20, 21]. Increasing evidence pinpoints the central role of systemic inflammation [22, 23] as well as higher vascular risk burden [24, 25] in Parkinson’s disease, a disorder characterized by progressive degeneration of the nigrostriatal dopamine system [26]. Previous work have further highlighted sex differences, suggesting that men have lower levels of dopaminergic markers [27–30], and possibly higher levels of inflammation [31–33]. To date, links between inflammation and dopamine integrity and potential sex differences have not been tested in the context of healthy aging. Furthermore, it is important to elucidate the relative contribution of inflammation and cerebrovascular dysfunction to dopamine decline.

During ongoing systemic inflammation, concentration of several proinflammatory markers is increased, including C-reactive protein (CRP) [34]. CRP levels are elevated in the acute phase of an infection or a trauma [35], but also show low, yet reliable, increases in older adults [36, 37], presumably reflecting ongoing low-grade inflammation. Chronic low-grade inflammation can trigger and perpetuate neuroinflammation via e.g., disruption of the blood-brain barrier and infiltration of inflammatory cells and proteins, upon which glial cells are activated [38–43], possibly leading to neurochemical changes, e.g., dopamine decline [44–50]. Meta-analyses of epigenome-wide association studies have shown that aging-related elevation of CRP levels are associated with DNA methylation levels at specific loci (CpG sites) in the genome [51, 52]. From these studies, aggregate methylation scores for CpGs associated with circulating CRP levels have been calculated and used as proxies for inflammation. Specifically, methylation at 58 independent CpG sites was associated with CRP levels in a sample composed of nine discovery (*n* = 8863) and four replication cohorts (*n* = 4111) [51]. Importantly, these CpG sites were found to be associated to CRP levels in independent samples [31, 53–55]. In a recent update in a larger sample (*n* = 22,774), 1,511 independent CpG sites associated with CRP levels were identified [52]. Previously, methylation-based scores of inflammation have been associated with measures of brain structure [56, 57], cognitive function [31, 56], and found to increase in response to upregulation of other inflammatory markers [55].

The main aim of the present work was to investigate whether inflammation scores at baseline (assessed via DNA methylation scores as proxies of inflammation) are associated with in vivo DRD2 availability in striatum (mean for caudate and putamen). We used one of the largest human in vivo dopamine studies: The Cognition, Brain, and Aging study (COBRA), in which 181 healthy, older adults had undergone positron emission tomography with ^11^C-raclopride to assess DRD2 availability [58]. Of these, 70% returned for a 5-year assessment. We expected individuals with higher inflammation scores at study baseline to have lower striatal DRD2 availability (Hypothesis 1a), and faster 5-year striatal DRD2 decline (Hypothesis 1b). Given previous research showing lower DRD2 levels [27–30] and higher inflammation burden in older men [31, 32], we hypothesized that inflammation-DRD2 associations would be stronger in men compared to women (Hypothesis 2). In previous work, we have shown that cerebral small-vessel disease, and specifically, white-matter lesion burden to be associated with individual differences in striatal DRD2 availability [4, 7]. Here, we assessed the respective contributions of lesions and inflammation scores to individual differences in striatal DRD2 availability. We expected lesion burden and inflammation scores to be intercorrelated, and higher lesion burden as well as higher inflammation scores to be associated with reduced DRD2 availability (Hypothesis 3).

## Methods

### Sample

Detailed descriptions of the a priori statistical power analysis, exclusion criteria, test protocols, prevalence of common aging-related disorders including frequency of cardiovascular risk factors, and drop-out between test waves, have been published previously [4, 58]. Briefly, at study onset, 181 healthy adults (100 men, 81 women) aged 64–68 years (mean 66.2 ± 1.2) were recruited after random selection from the population registry in Umeå (Sweden). Exclusion criteria were neurological and psychiatric disorders, previous brain trauma, intellectual disability, a Mini-Mental State Examination score < 27, structural brain abnormalities, cancer, diabetes, severe auditory and visual impairments, claustrophobia, and metal implants. Inflammatory disorders were not exclusion criteria and noted in 17 individuals [58]. A 5-year follow-up was conducted with 129 returnees (69 men, 60 women), now aged 69–73 years (mean 71.2 ± 1.2), where all assessments, apart from blood sampling, were repeated.

### DRD2 availability

The same scanners were used at both time points. Specifically, MRI scanning was conducted using a 3 Tesla Discovery MR 750 scanner (General Electric, Milwaukee, WI, USA) and PET data were collected using a Discovery PET/CT 690 system (General Electric).

To estimate DRD2 availability (binding potential; BP_ND_), a 55-min, 18-frame (9 × 120 s + 3 × 180 s + 3 × 260 s + 3 × 300 s) dynamic PET scan was acquired during rest following intravenous bolus injection of approximately 250 MBq ^11^C-raclopride (baseline: 263.5 ± 19.0 MBq; follow-up: 260.2 ± 15.0 MBq). A CT scan (20 mA, 120 kV, 0.8 s/revolution) preceded ligand injection. Attenuation- and decay-corrected images (47 slices, field of view = 25 cm, 256 × 256-pixel transaxial images, voxel size = 0.977 × 0.977 × 3.27 mm^3^) were reconstructed with the iterative algorithm VUE Point HD-SharpIR (GE; 6 iterations, 24 subsets, 3.0 mm post filtering; FWHM: 3.2 mm). PET images were motion-corrected and co-registered with T1-weighted images from the corresponding timepoint (baseline or follow-up) using the Statistical Parametric Mapping software (SPM12). The mean of the first five frames was used as source for image co-registration. Three participants did not undergo MRI at follow-up, and therefore, PET images from both time points were co-registered with the baseline T1-image. Two individuals declined to undergo PET at follow-up (total *n* = 127).

Regions of interest (ROIs) for the DRD2 analysis were caudate and putamen. ^11^C-raclopride BP_ND_ was estimated with correction for partial-volume effects (PVE). This was achieved via regional PVE correction using the symmetric geometric transfer-matrix implemented in FreeSurfer. Initial correction was achieved using resolution modelling in the iterative image reconstruction procedure (SHARP-IR), and the remnant PVE was controlled for using the ROI-based geometric transfer matrix approach. The size of the secondary correction kernel was estimated empirically (point-spread function = 2.5 mm; isotropic), to achieve a similar level of correction as earlier [59]. FreeSurfer segmentations and pre-processed PET data were used to estimate PVE-corrected regional radioactivity concentrations per ROI and time frame. PVE-corrected BP_ND_ estimates were calculated with a multilinear reference-tissue model on dynamic PVE-corrected data, with the cerebellar grey matter as reference region.

### White matter lesions

White matter lesions were identified as hyperintensities in fluid-attenuated inversion recovery (FLAIR) images (48 slices, slice thickness = 3 mm, TE = 120 milliseconds, TR = 8,000 milliseconds, field of view = 24 cm). The segmentation was performed using the lesion-growth algorithm [60] available in the LST toolbox version 2.0.14 for SPM12. T1-weighted images were segmented into three main tissue classes: cerebrospinal fluid, grey matter, and white matter, and co-registered with the FLAIR intensities to calculate lesion-belief maps. Maps were thresholded (κ = 0.3) to obtain initial binary lesion map, which were then grown along hyperintense neighboring voxels in the FLAIR image, and thresholder at 50% to yield a binary lesion map. From these calculations, total lesion volume (ml) was estimated.

### Lifestyle and health

Health variables, known disorders, and medications were documented [58]. Blood pressure was measured in sitting position. From this information, a composite risk score was calculated, estimating the probability of 10-year cardiovascular disease event using the algorithm from the Framingham Heart Study [61]. Variables that were entered into the composite score included hypertension, systolic blood pressure, BMI, smoking status, age, and sex.

### DNA methylation analyses

DNA methylation is an epigenetic marker characterized by the addition of a methyl group to cytosines followed by guanines, referred to as CpG dinucleotides [62]. CpGs are found in CpG islands located near gene promoters where methylation is linked to gene expression levels [63]. The methylation analyses were performed at Bioinformatics and Expression Analysis core facility (BEA; Karolinska Institute, Sweden). 500 ng of genomic DNA was bisulfite converted with the EZ-96 DNA Methylation kit (Zymo Research, Irvine, CA, USA) and genome-wide DNA methylation analysis was performed using the Infinium MethylationEPIC BeadChip (Illumina, San Diego, CA, USA, 2020). The array was designed for genome wide methylation analysis and covers > 850 000 methylation sites at single nucleotide resolution. Selection of genes was originally guided by literature, and primarily, from the Gene Ontology project (geneontology.org). As such, it covers CpG islands, shores, shelves, and the promoted genes. The technique uses two different probe types (Infinium 1 and 2) with different characteristics, thus requiring normalization to reduce technical bias.

For analysis, visualization, and extraction of methylation data, the GenomeStudio software version 2011.1 (Illumina Inc.) was used. The protocol combined bisulfite conversion of genomic DNA and whole-genome amplification with direct, array-based capture and scoring of the CpG loci. Allele-specific single base extension of the probes incorporated a biotin nucleotide, or a dinitrophenyl-labeled nucleotide. Signal amplification of the incorporated label further improved the overall signal-to-noise ratio of the assay. Signal intensity was measured to generate *β*-values, i.e., the degree of methylation at a locus (0 = no methylation; 1 = 100% methylation). *β*-values were estimated as the ratio of signal intensity of the methylated alleles to the sum of methylated and unmethylated intensity signals.

DNA samples were divided over several arrays when performing the methylation analyses. Principal component analysis revealed that one of the total six batches had *β*-values that were significantly higher as compared to the other batches. In order to combine data from all batches, we adjusted the methylation values using the ComBat method in R [64]. This method is robust when it comes to data harmonization, even when sample sizes are relatively small. Note that in the supplementary material, we also report analyses performed with unadjusted values, from which the batch with elevated values (*n* = 37) were excluded (effective sample: *n* = 144; see also [65]). Furthermore, blood cell counts (granulocytes, natural killer (NK) cells, B-cells, CD4 + T-cells, and CD8 + T cells) were estimated from the DNA methylation array using the Houseman method [66] in *minfi* [67, 68]. In all analyses, NK cells and granulocytes were entered as covariates, as these were significantly associated with the inflammation scores (*r*-values between − 0.37 and 0.49, *p*-values < 0.05).

### Inflammation scores

**Fig. 1:**
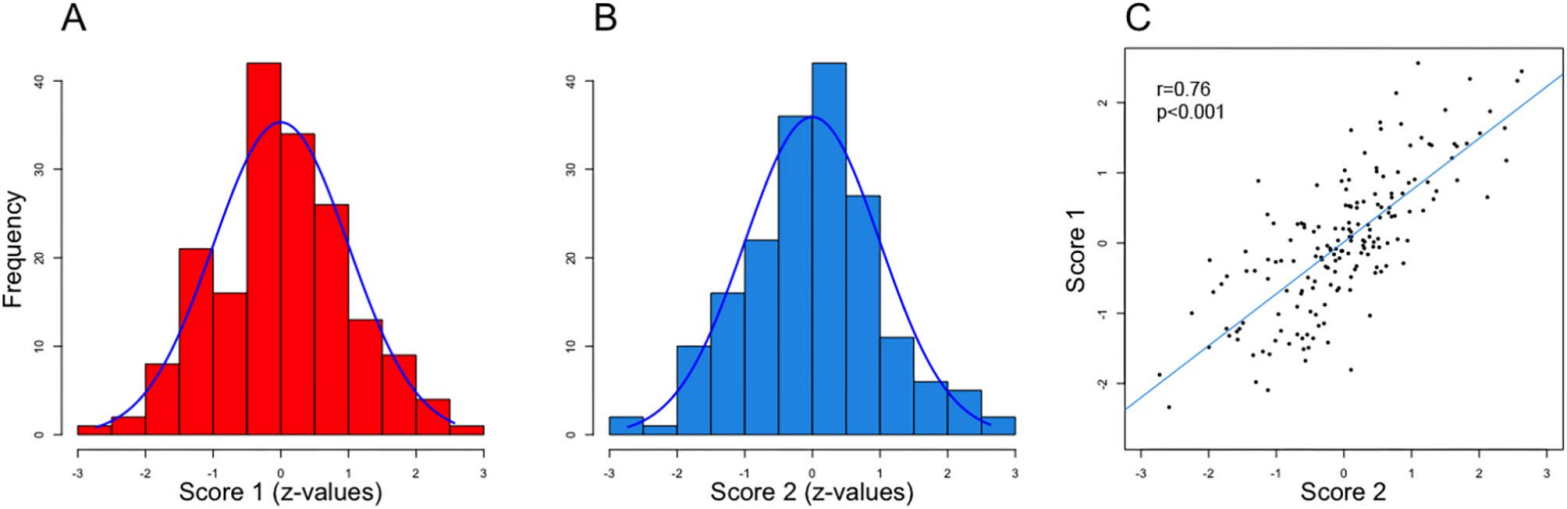
DNA methylation scores as proxies for peripheral inflammation. **A**: Histogram for score 1 (defined by Ligthart et al., 2016). **B**: Histogram for score 2 (defined by Wielscher et al., 2022). **C**: Correlation between scores 1 and 2

DNA methylation of previously defined CpG sites were used to calculate two separate scores that have been associated with CRP levels [51, 52]. Importantly, methylation of these select CpG sites and links to inflammation have been replicated by other studies [31, 55, 56], indicating the reliability of these scores in representing inflammatory levels. The two scores included 58 CpG sites [51] and 1511 CpG sites [52], respectively. Scores were calculated by multiplying *b*-values in our sample for each CpG site with weights reported by authors originally defining these scores. Then, values per CpG were summarized and z-transformed to create standardized composite scores. In the present work, data were missing for a few CpG sites (9 out of 58 CpGs for score 1 [51]; 28 of 1511 CpG for score 2 [52]). Scores were normally distributed (*score 1*: skewness = 0.07 and kurtosis = 2.94; *score 2*: skewness = 0.05 and kurtosis = 3.24; see Fig. 1A, B) and were also intercorrelated (*r* = 0.76, *p* < 0.001; Fig. 1C).

### Statistical analyses

All data analyses were conducted using the R software [69] (Version 4.2.2) and the R Studio [70] integrated development environment for R (Version: 2023.06.0 + 421). Univariate outliers were defined as values ± 3.29 standard deviations (*SD*) from the mean [71] and were excluded as pairwise deletions. Methylations scores were, however, excluded as listwise deletions in case at least one CpG was an outlier (*n* = 4 for score 1; *n* = 1 for score 2). Multivariate outliers for DRD2 values between striatal regions (caudate and putamen) and methylation scores were identified according to the Mahalanobis distance (*p* < 0.001) and excluded as listwise deletions (*n* = 2 for DRD2; *n* = 0 for scores 1 and 2). The final sample used in the analyses ranged between 172 and 180 at baseline, and between 118 and 124 for estimates of DRD2 change. Following exclusions, normal distributions were observed for all variables (skewness: -0.24 to 0.57; kurtosis: 2.64 to 3.37) except for lesion volumes (skewness: 1.86 to 2.04; kurtosis: 6.75 to 6.89). Therefore, lesion volumes were log-transformed before statistical analyses (skewness: -0.79 to -0.50; kurtosis: 3.45 to 3.68). Descriptive data are reported as mean values and SDs, or as estimated marginal means and standard errors following cell correction. The significance level for all testing was 5% and *p*-values were corrected for multiple comparisons using Bonferroni correction.

The inflammation-DRD2 link (hypothesis 1) was assessed via multiple linear regression models. Following the assumption that change was not systematically linked to baseline levels [4], DRD2 change (%) over 5 years was calculated as:

$$\:Change\:\left(\%\right)=\frac{Follow\:up-baseline}{baseline}\bullet\:100$$

Sex differences in the inflammation-DRD2 link (hypothesis 2) were assessed by including the inflammation score by sex interaction term into the models. Differences in the inflammation-DRD2 link were also assessed via partial correlations at the sample level, and for men and women separately. The significance of the differences for these correlations was then compared between groups (men versus women) using a two-tailed z-test [72]. Sex differences in inflammation scores, and other between-group differences, were assessed with analysis of covariance (ANCOVA) or independent samples t-tests. Relationships among inflammation scores, cardiovascular-related risk factors, and white-matter lesion burden were evaluated via partial correlations. Furthermore, multiple regression models were used to test whether baseline inflammation scores and white-matter lesions independently predicted DRD2 availability when entered into the same model (hypothesis 3). All analyses were adjusted for cell count and age, as these may influence the inflammation scores and DRD2 levels.

## Results

### Relationships between inflammation scores and *in vivo* DRD2 availability

**Fig. 2:**
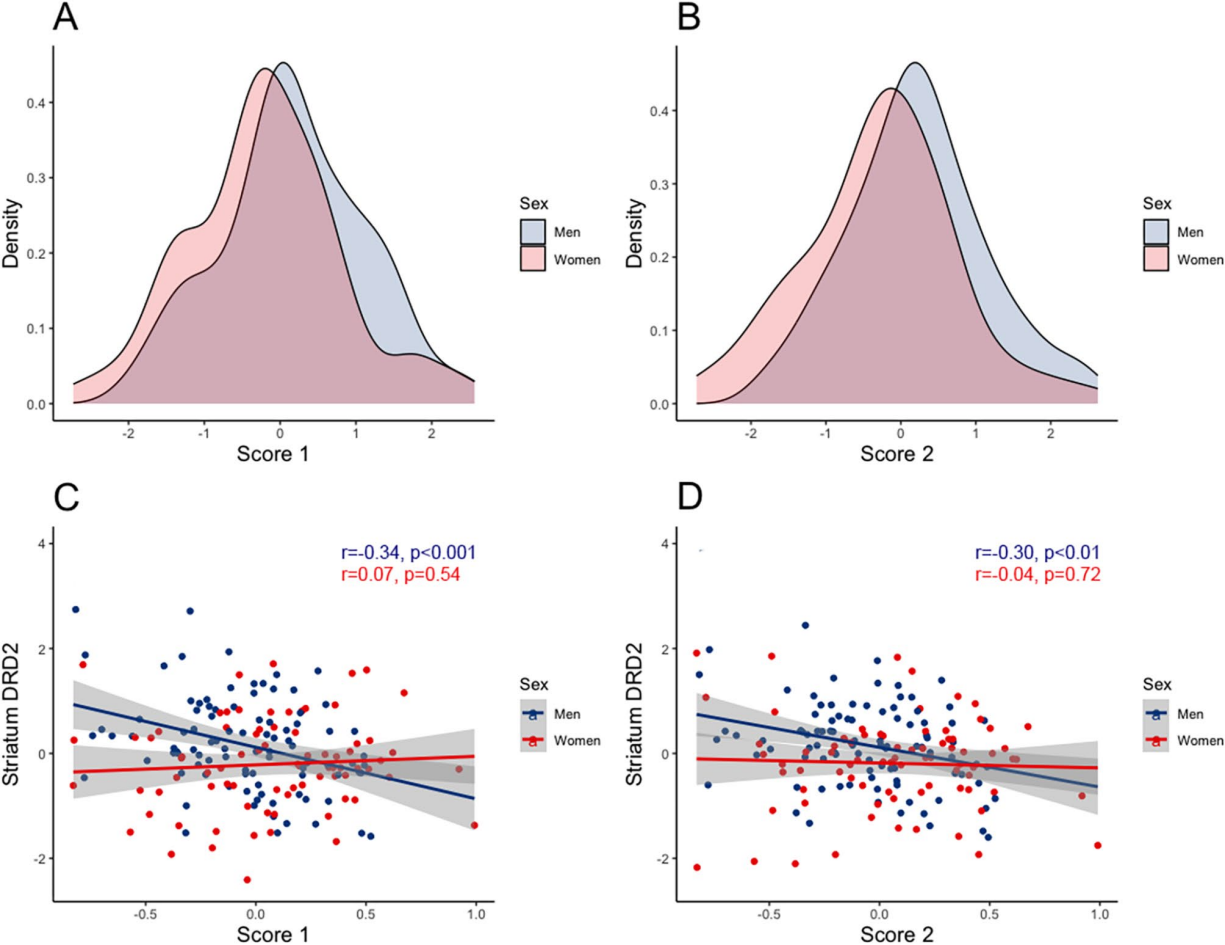
Association between inflammation scores and D2-receptor (DRD2) availability. **A** and **B**: density plots for inflammation scores for men (blue) and women (pink). C and D: partial correlations (adjusted for cell count and age) between inflammation scores and striatal DRD2 availability (mean for putamen and caudate) for men (blue) and women (red)

We first assessed the relationship between inflammation scores (mean values = 0, *SDs* = 1) and striatal DRD2 availability (mean BP_ND_ value at baseline = 3.26, *SD* = 0.36; mean BP_ND_ value at follow-up = 3.15, *SD* = 0.37; mean 5-year change (%) = − 1.91, *SD* = 6.98; hypothesis 1a). Multiple regression models for the whole sample were not statistically significant (models 1 and 2 in Table 1). We then went on to assess sex differences in this association via ANCOVA (hypothesis 2). In line with previous studies [31–33], men had higher inflammation scores than women (*score 1*: 0.19±0.09 for men and − 0.24± 0.11 for women, *F* = 8.23, *p* < 0.01; *score 2*: 0.17±0.09 for men and − 0.21±0.10 for women, *F* = 16.44, *p* < 0.001; Fig. 2A, B). In multiple regression models 3 and 4, main effects of inflammation scores, as well as sex by inflammation score interactions, were found in relation to striatal DRD2 availability (Table 1). To illustrate what the interaction term conveys, scatter plots for residuals adjusted for age and cell count are shown for men and women, separately, (Fig. 2C, D). These demonstrate that associations between both inflammation scores and striatal DRD2 levels are observed for men, but not for women. Notably, correlations for men versus women were statistically different (*score 1*: z = − 2.71, *p* < 0.01; *score 2*: z = − 1.74, *p* < 0.05 for two-tailed z-test). Importantly, largely similar results were observed when performing the analyses with the two DNA methylation scores without adjustment for batch effects, where the batch with elevated methylation values were instead excluded (see supplementary material). Individuals with inflammatory disorders (*n* = 17) had higher inflammation scores, as compared to those without (independent samples t-test: t(174) = 2.4, *p* < 0.05 for score 1; t(177) = 1.8, *p* = 0.07 for score 2). No differences were found for distribution of men and women in groups with versus without inflammatory disorders (*p* > 0.05 for chi-squared test).

**Table 1:** Regression models for inflammation scores in relation to baseline striatal D2-receptor availability for the whole sample

	Variable	*β*	t	*p*
***Model 1:*** F (4, 166) = 1.78 *p* = 0.14 *R*^2^ = 0.04 Adjusted *R*^2^ = 0.02	Inflammation score 1 NK Granulocytes Age	−0.18 0.04 −0.16 −0.04	−2.20 0.47 −1.83 −0.51	0.03 0.64 0.07 0.61
***Model 2:*** F (4, 169) = 2.25 *p* = 0.07 *R*^2^ = 0.05 Adjusted *R*^2^ = 0.03	Inflammation score 2 NK Granulocytes Age	−0.23 0.11 −0.14 −0.03	−2.55 1.11 −1.67 −0.44	0.01 0.27 0.10 0.66
***Model 3***: F (6, 164) = 3.24 *p* < 0.01 R^2^ = 0.11 Adjusted R^2^ = 0.07	Inflammation score 1 Sex NK Granulocytes Age Sex⋅score 1	−0.33 0.38 0.12 −0.07 −0.03 0.41	−3.07 2.30 1.28 −0.79 −0.34 2.71	**< 0.01** 0.02 0.20 0.43 0.74 **< 0.01**
***Model 4***: F (6, 167) = 3.04 *p* < 0.01 R^2^ = 0.10 Adjusted R^2^ = 0.06	Inflammation score 2 Sex NK Granulocytes Age Sex⋅score 2	−0.37 0.34 0.18 −0.07 −0.02 0.34	−3.08 2.04 1.83 −0.83 −0.33 2.25	**< 0.01** 0.04 0.07 0.41 0.74 0.03

Main effects of inflammation scores (models 1, 2) and interactions between sex and inflammation scores (models 3, 4) are shown in relation to striatal D2-receptor availability (average for putamen and caudate). Covariates in all models are age and cell count (NK and granulocytes). Values in bold font survived Bonferroni adjustment (*p* = 0.05/4 for models 1 and 2, and *p* = 0.05/6 for models 3 and 4)

We then assessed associations between inflammation scores at baseline and 5-year striatal DRD2 change (%; hypothesis 1b). Multiple regression models for the whole sample were not significant (score 1: *F* (4, 115) = 1.05, *p* = 0.38; score 2: *F* (4, 116) = 0.68, *p* = 0.61), indicating that individual differences in inflammation levels at study entrance did not predict DRD2 change over this period. Selective attrition may influence longitudinal findings. Here, drop-outs and returnees were comparable in terms of inflammation scores (independent samples t-test: *t* (113, 4) = − 0.52, *p* = 0.61), and as previously shown, DRD2 levels were slightly higher for drop-outs [4].

### Inflammation scores and vascular health as predictors of DRD2 availability

Inflammation scores were tested for associations with vascular factors (see partial correlations in Table 2). Mean values were 2.46 ± 2.41 ml for lesion volumes, 24.11 ± 10.80% for a composite score of 10-year CVD risk (i.e., the Framingham risk score), 26.01 ± 3.36 for BMI, 141.80 ± 17.39 mmHg for systolic blood pressure, and 85.04 ± 9.82 mmHg for diastolic blood pressure. These analyses showed that both inflammation scores were associated with the 10-year CVD risk. As expected, a higher composite score was linked to higher inflammation scores. While correlations were also found with one of the inflammation scores in relation to BMI, this association did not survive Bonferroni adjustment. Furthermore, no correlations were found between the inflammation scores and chronological age or white-matter lesion burden. We then performed multiple regression analyses to test the relative contributions of inflammation and white-matter lesions to DRD2 availability (hypothesis 3). When considering the whole sample, lesions, but not inflammation scores, were significantly associated with striatal DRD2 availability (Table 3). In separate regression models for men and women, white-matter lesions load as well as inflammation scores were significant predictors of striatal DRD2 availability in men, but not in women. Only the association between inflammation scores and DRD2 availability survived Bonferroni correction.

**Table 2:** Associations between inflammation scores and health-related measures

	Score 1 (based on 58 CpGs)	Score 2 (based on 1511 CpGs)
Age White matter lesions (ml) 10-year CVD risk (%) BMI Systolic bp (mmHg) Diastolic bp (mmHg)	−0.07 0.07 **0.27** ^***^ 0.17^*^ 0.08 0.04	−0.06 0.06 0.17^*^ 0.06 0.00 −0.01

Partial correlations (adjusted NK and granulocyte count and age) are reported with Pearson’s correlation coefficient (*r*). ^*^p < 0.05, ^***^p < 0.001. The value in bold survived Bonferroni adjustment (*p* = 0.05/6). 10-year CVD risk refers to the Framingham risk score. Abbreviations include CVD: cardiovascular disease, BMI: body-mass index, bp: blood pressure

**Table 3:** Associations among inflammation scores, white-matter lesions, and striatal D2-receptor availability

	Variable	*β*	t	*p*
**Whole sample** *Model 1*: F (5, 156) = 2.79 *p* = 0.02^*^ *R*^2^ = 0.08 Adjusted *R*^2^ = 0.05 *Model 2:* F (5, 159) = 2.94 *p* = 0.01^*^ *R*^2^ = 0.08 Adjusted *R*^2^ = 0.06	Inflammation score 1 White-matter lesions NK Granulocytes Age Inflammation score 2 White-matter lesions NK Granulocytes Age	−0.15 −0.2 0.03 −0.15 0.03 −0.17 −0.21 0.07 −0.11 0.03	−1.86 −2.66 0.35 −1.6 0.33 −1.88 −2.75 0.75 −1.33 0.33	0.07 **< 0.01** 0.72 0.11 0.74 0.06 **< 0.01** 0.45 0.19 0.74
**Men** *Model 3*: F (5, 83) = 3.32 *p* = 0.00^**^ *R*^2^ = 0.17 Adjusted *R*^2^ = 0.12 *Model 4*: F (5,85) = 2.87 *p* = 0.02^*^ *R*^2^ = 0.14 Adjusted *R*^2^ = 0.09	Inflammation score 1 White-matter lesions NK Granulocytes Age Inflammation score 2 White-matter lesions NK Granulocytes Age	−0.33 −0.20 −0.01 −0.21 −0.07 −0.34 −0.21 0.11 −0.11 −0.06	−3.18 −2.07 −0.07 −1.66 −0.73 −2.95 −2.19 0.85 −0.83 −0.64	**< 0.01** 0.04 0.94 0.10 0.47 **< 0.01** 0.03 0.4 0.37 0.52
**Women** *Model 5*: F (5, 67) = 1.49 *p* = 0.21 R^2^ = 0.10 Adjusted R^2^ = 0.03 *Model 6*: F (5, 68) = 1.63 *p* = 0.16 R^2^ = 0.11 Adjusted R^2^ = 0.04	Inflammation score 1 White-matter lesions NK Granulocytes Age Inflammation score 2 White-matter lesions NK Granulocytes Age	0.09 −0.18 0.16 0.00 0.13 0.06 −0.20 0.13 −0.07 0.15	0.71 −1.48 1.22 0.00 1.10 0.43 −1.68 0.91 −0.51 1.26	0.48 0.14 0.23 1.00 0.28 0.67 0.10 0.37 0.61 0.21

Regression models were conducted for the whole sample (models 1, 2), and in men (models 3, 4) and women (models 5, 6) separately. Striatal D2-receptor availability represents an average for putamen and caudate. Covariates in all models are age and NK and granulocyte count

Values in bold survived Bonferroni adjustment (*p* = 0.05/5)

## Discussion

Healthy aging is characterized by decreased striatal dopamine markers, including dopamine receptors [3, 4] and dopaminergic neurons in the substantia nigra [73, 74]. Inflammation, when induced via endotoxins in mouse models, or observed in patients with Parkinson’s disease, has been associated with progressive nigrostriatal dopamine degeneration [22, 23, 75, 76]. Although not formally tested previously, the link between aging-related chronic inflammation and dopamine system integrity has been suggested to result in behavioral malfunction, such as cognitive decline and depression [2, 44, 77, 78]. The present study provides initial support for associations between one proxy of inflammation and individual differences in striatal (caudate and putamen) DRD2 availability in healthy, older men, but not with 5-year DRD2 decline rates. The inflammation score-DRD2 link was selectively found for men, who also showed lower DRD2 levels [27] and higher inflammation scores.

DNA methylation patterns undergo profound alterations across the human lifespan and are suggested to be a significant component of the aging process [63]. Interindividual variation in DNA methylation patterns may reflect the interplay of environmental factors, such as infections, disease burden, and lifestyle habits, but also random events and genetically driven mechanisms [79]. The current work used DNA methylation scores as proxies of inflammation. These scores have been estimated in large-sized samples [51, 52] and replicated in terms of associations to inflammatory markers [53] and behavioral parameters [31, 54]. While the present study cannot compare the inflammation scores in relation to other inflammatory markers, we did find that both inflammation scores were associated with CVD risk (specifically the Framingham risk score), and one of them with BMI. The link between inflammation and CVD risk has been replicated over studies [17–19], and meta-analyses show that a large portion of CRP-related CpGs were significantly associated with BMI [51, 52]. The inflammation-DRD2 associations reported here may be underestimated due to missingness of some of the CpGs that are part of these scores. Despite this, and the unique variance between the two inflammation scores (about 40%), similar associations were found for both scores and DRD2 availability. This speaks for robustness of these findings.

Inflammatory markers originating from the periphery, such as cytokines, have access to the brain via e.g., leaky regions of the blood-brain barrier and afferent fibers of the vagus nerve [22, 80–82]. Infiltration of peripheral inflammatory markers is linked to glial cell activation—a hallmark of neuroinflammation [38–41]. While acute neuroinflammation has neuroprotective effects, persistent or chronic neuroinflammation is associated with neurodegeneration [43, 46, 47]. Hence, dopamine degeneration may follow chronic inflammation. Still, the relationship between inflammation and dopamine decline may be reciprocal, where dopamine loss further propagates inflammation [83]. To exemplify, activation of DRD2s was found to exert anti-inflammatory effects [84–86]. Higher DNA-methylation scores have in some studies been associated with white-matter lesions burden, but with inconsistencies across studies [87–90]. The absence of an inflammation score-lesion association in the current work may reflect good health and the relatively low lesion burden in the COBRA sample. Possibly, associations are more evident when considering higher cerebral small-vessel disease progression, larger age ranges, and longer durations of time. Longitudinal observations over decades did indeed suggest that increasing and persistent systemic inflammation after midlife may promote white matter pathology in older populations [91]. Previously, we showed that men in the COBRA sample have higher lesion load, reduced grey-matter perfusion, and lower striatal DRD2 availability, as compared to women [27]. The present work further shows that men are further characterized by higher inflammation scores, which together with white-matter lesions serves as two sources of individual differences in DRD2 integrity in aging.

Previous studies have reported higher CRP levels in older adults, and selectively in men [32], while others did not find such sex differences [92]. CRP-associated DNA methylation scores have been found to increase with age, and more rapidly so in men than in age-matched women [31]. Other studies focusing on epigenetic clocks have also found that males have accelerated biological aging, as compared to females [93, 94]. In relation to the findings presented in the current work, it remains to be elucidated whether men are more susceptible to dopamine decline, e.g., because of higher white-matter lesion load [27] and inflammatory processes, or if other factors are at play. We did not find support for an association between higher inflammation scores and 5-year striatal DRD2 losses, neither when considering the whole sample nor in men and women separately. Possibly, exposure to inflammation over longer time spans may give rise to individual differences in dopamine integrity, yet other factors may be more prominent in driving dopamine decline over shorter durations of time, such as progression of cerebrovascular insults [4]. Blood sampling was not conducted at the follow-up session; hence we were not able to assess whether *changes* in inflammation scores and DRD2 availability are correlated. We further note that effect sizes for associations may be underestimated due to the strict exclusion criteria and high health of the COBRA sample. Possibly, larger effects may be observed when studying samples with higher frequencies of inflammatory diseases (a group for which inflammatory scores were elevated in the current study), but also, frequencies of common aging-related disorders that are representative of the average population. To exemplify, prevalence of hypertension and hyperlipidemia was 33% and 16% in COBRA [58] versus ∼50% and 30% for those aged 60–64 years in the region where COBRA was conducted (source: socialstyrelsen.se). Obesity in the COBRA sample and those born during the 1940s in the general Swedish population were, however, comparable and ∼15% (source: folkhälsomyndigheten.se).

## Conclusions

This work indicates a link between one proxy of inflammation and reduced dopamine system integrity in aging, and selectively in men. The impact of inflammation scores and vascular lesions were largely comparable (effect-size wise), pointing at two potential targets that may be of interest in future intervention studies. The use of DNA methylation-based scores as proxies for inflammation may not capture the intricate nature of inflammatory mechanisms, so future studies should assess the links between other inflammatory markers and dopamine system integrity. Another limitation pertains to the study sample size, which despite being large in the context of PET imaging [3] still might impose limitations on detecting subtle effects, particularly when stratified by sex. Future work should assess (i) whether the observed sex-specific link translates to other inflammatory and neuroinflammatory markers, particularly in subregions of the striatum for the latter, (ii) whether there are potential modulators of the dopamine-inflammation link, and (iii) the implications of inflammation-related dopaminergic reductions for cognitive decline and reward-processing in aging.

## Electronic supplementary material

Below is the link to the electronic supplementary material.

Supplementary Material 1

## Data Availability

The data sets generated and analyzed are available from the corresponding author upon reasonable request from a qualified investigator. Prerequisites encompass a formal data sharing agreement, approval of a formal project outline, and ethical permission for the outlined research questions.

## Abbreviations

DRD2: DA D2-like receptor
PET: Positron emission tomography
MRI: Magnetic resonance imaging
CRP: C-reactive protein
DNA: Deoxyribonucleic acid
CpG: Cytosine nucleotide followed by a guanine nucleotide
COBRA: Cognition, brain, and aging
NK-cell: Natural killer cell
CVD: Cardiovascular disease
BMI: Body-mass index
BP_ND_: Binding potential
