# Supplementary material for: Associations between inflammation and striatal dopamine D2-receptor availability in aging
Source: J Neuroinflammation. 2025 Jan 30;22:24. doi: 10.1186/s12974-025-03355-0 (PMC11783874; doi:10.1186/s12974-025-03355-0)
Supplement: Supplementary file 1 — Supplementary Material 1 [file 12974_2025_3355_MOESM1_ESM.pdf]

Supplementary material

Figure 1. DNA methylation scores as proxies for peripheral inflammation. A: Histogram for score 1 (defined by Ligthart et al., 2016). B: Histogram for score 2 (defined by Wielscher et al., 2022). C: Correlation between scores 1 and 2.

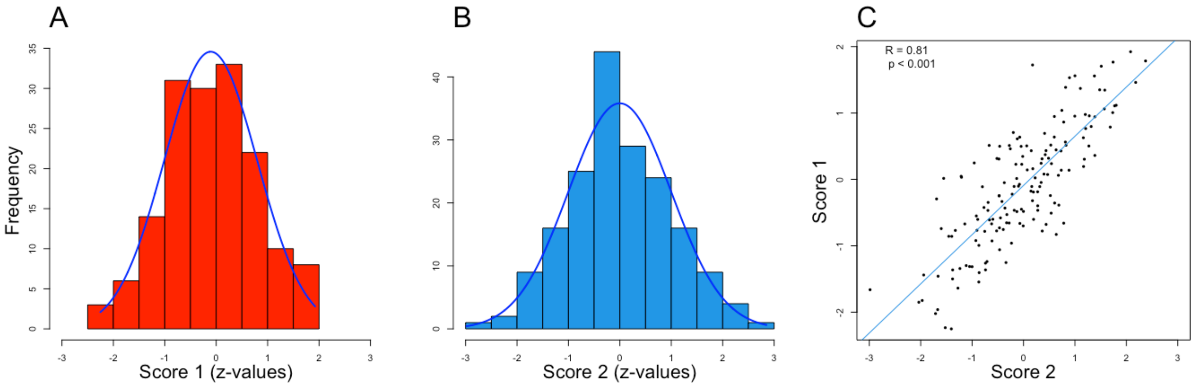

Figure 2. Association between inflammation and D2-receptor (DRD2) availability. Density plots for inflammation scores 1 (A) and 2 (B) are shown for men (blue) and women (pink). C and D: Partial correlations (adjusted for cell count and age) between inflammation scores and striatal DRD2 availability (mean levels in putamen and caudate) for men (blue) and women (red).

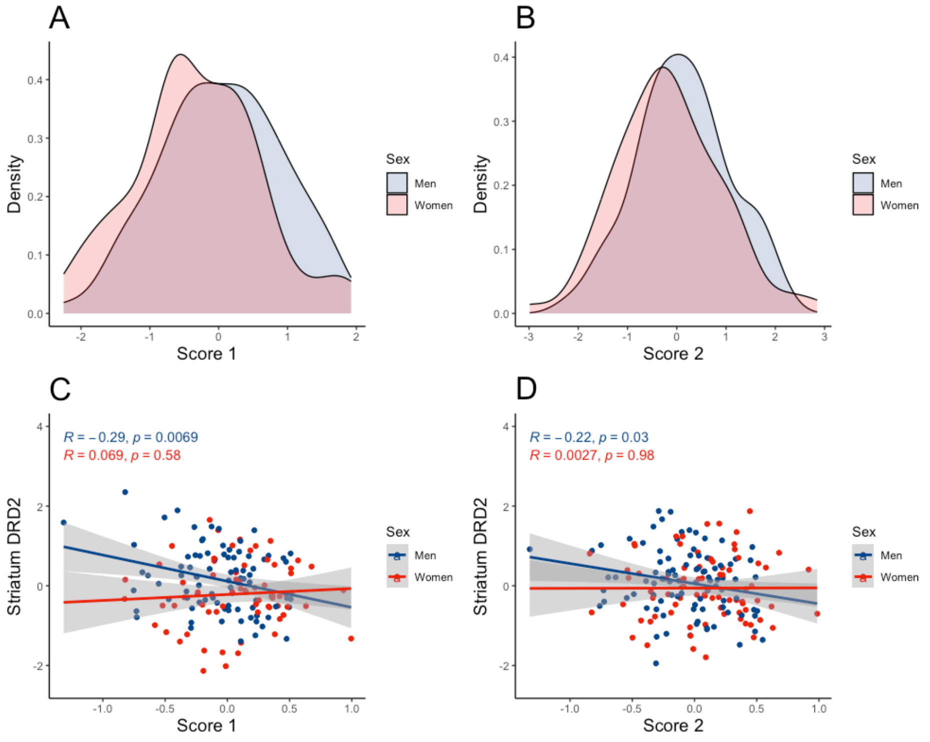

Table 1. Regression models for inflammation scores in relation to baseline striatal D2-receptor availability. Methylation values are not adjusted with ComBat, but instead, the batch with elevated methylation values (*n*=37) was excluded.

	**Variable**	***β***	**t**	***p***
Model 1:				
F(4,119)=0.80	Inflammation score 1	-0.15	-1.45	0.15
p=0.53	NK	-0.03	-0.31	0.75
R^2=0.03	Granulocytes	-0.16	-1.40	0.16
adjusted R^2=-0.01	Age	-0.02	-0.20	0.85
Model 2:				
F(4,134)=0.84	Inflammation score 2	-0.13	-1.29	0.20
p=0.50	NK	-0.01	-0.06	0.95
R^2=0.02	Granulocytes	-0.15	-1.54	0.13
adjusted R^2=0.00	Age	0.00	-0.04	0.97
Model 3:				
F(6,117)=2.50	Inflammation score 1	-0.28	-2.12	0.04
p=0.03	Sex	0.51	2.61	**0.01**
R^2=0.11	NK	0.07	0.64	0.52
adjusted R^2=0.07	Granulocytes	-0.05	-0.46	0.65
	Age	-0.01	-0.06	0.95
	Sex·score 1	0.41	2.32	0.02
Model 4:	Inflammation score 2	-0.30	-2.16	0.03
F(6,132)=2.39	Sex	0.47	2.61	**0.01**
p=0.03	NK	0.12	1.07	0.28
R^2=0.10	Granulocytes	-0.07	-0.65	0.52
adjusted R^2=0.06	Age	0.00	0.02	0.98
	Sex·score 2	0.35	2.06	0.04

Main effects of inflammation (models 1, 2) and interactions between sex and inflammation (models 3, 4) are shown in relation to striatal D2-receptor availability (average for putamen and caudate). Covariates in all models are age and cell count (NK and granulocytes). Values in bold font survived Bonferroni adjustment (p = 0.05/6 for models 3 and 4).

Table 2. Associations between inflammation scores and health-related measures. Methylation values are not adjusted with ComBat, but instead, the batch with elevated methylation values (*n*=37) was excluded.

	Score 1 (based on 58 CpGs)	***Score 2*** (based on 1511 CpGs)
Age	-0.04	0.03
White matter lesions (ml)	0.08	0.09
10-year CVD risk (%)	0.22*	0.10
BMI	0.14	0.08
Systolic bp (mmHg)	-0.04	-0.01
Diastolic bp (mmHg)	0.05	0.03

Note. Values represent the Pearson's correlation coefficient (*r*). Associations represent partial correlations and are adjusted for cell count (NK and granulocytes) and age. 10-year CVD risk: * $p < 0.05$. No associations survived Bonferroni adjustment ($p = 0.05/6$). Abbreviations include CVD: cardiovascular disease, BMI: body-mass index, bp: blood pressure.

Table 3. Multiple regression models of inflammation scores and white-matter lesions in relation to striatal D2-receptor availability. Methylation values are not adjusted with ComBat, but instead, the batch with elevated methylation values (*n*=37) was excluded.

	Variable	*β*	*t*	*p*
**Whole sample**				
Model 1:	Inflammation score 1	-0.13	-1.38	0.17
F(5,110)=2.00	White-matter lesions	-0.23	-2.46	0.02
p=0.08	NK	-0.04	-0.38	0.70
R^2=0.08	Granulocytes	-0.15	-1.27	0.21
adjusted R^2=0.04	Age	0.02	0.19	0.85
Model 2:	Inflammation score 2	-0.07	-0.73	0.47
F(5,123)=1.75	White-matter lesions	-0.21	-2.44	0.02
p=0.13	NK	-0.02	-0.20	0.84
R^2=0.07	Granulocytes	-0.11	-0.12	0.27
adjusted R^2=0.03	Age	0.03	0.40	0.69
**Men**				
Model 3:	Inflammation score 1	-0.33	-2.64	0.01
F(5,61)=1.72	White-matter lesions	-0.12	-1.00	0.32
p=0.14	NK	0.02	0.15	0.88
R^2=0.12	Granulocytes	-0.18	-1.09	0.28
adjusted R^2=0.05	Age	-0.10	-0.89	0.38
Model 4:	Inflammation score 2	-0.25	-1.78	0.08
F(5,66)=1.08	White-matter lesions	-0.14	-1.26	0.21
p=0.38	NK	0.10	0.62	0.54
R^2=0.08	Granulocytes	-0.09	-0.61	0.55
adjusted R^2=0.01	Age	-0.08	-0.75	0.46
**Women**				
Model 5:	Inflammation score 1	0.22	1.41	0.17
F(5,43)=2.13	White-matter lesions	-0.39	-2.58	0.01
p=0.08	NK	0.01	0.09	0.93
R^2=0.20	Granulocytes	-0.06	-0.33	0.74
adjusted R^2=0.11	Age	0.21	1.40	0.17
Model 6:	Inflammation score 2	0.14	0.94	0.35
F(5,51)=1.90	White-matter lesions	-0.27	-2.13	0.04
p=0.11	NK	0.02	0.13	0.90
R^2=0.16	Granulocytes	-0.10	-0.63	0.53
adjusted R^2=0.07	Age	0.22	1.57	0.12

Associations to striatal D2-receptor availability (average for putamen and caudate) are shown for the whole sample (models 1, 2), men (models 3,4) and women (models 5,6). Covariates in all models are age and NK and granulocyte count. No values survived Bonferroni adjustment (p = 0.05/5).
